# Procalcitonin biomarker kinetics fails to predict treatment response in perioperative abdominal infection with septic shock

**DOI:** 10.1186/cc13082

**Published:** 2013-10-24

**Authors:** Boris Jung, Nicolas Molinari, Mourad Nasri, Zied Hajjej, Gerald Chanques, Helene Jean-Pierre, Fabrizio Panaro, Samir Jaber

**Affiliations:** 1 Intensive Care Unit, Department of Anaesthesia and Critical Care, University of Montpellier, Saint Eloi Teaching Hospital, 80 avenue Augustin Fliche, F-34295 Montpellier, Cedex 5, France; 2 Institut National de la Santé et de la Recherche Médicale Unit 1046 (INSERM U-1046), Université Montpellier 1, Université Montpellier 2, 80 avenue Augustin Fliche, F-34295 Montpellier, Cedex 5, France; 3 DIM, UMR 729 MISTEA, La Colombière Teaching Hospital, 80 avenue Augustin Fliche, F-34295 Montpellier, Cedex 5, France; 4 Department of Microbiology, University of Montpellier, Arnaud de Villeneuve Teaching Hospital, 80 avenue Augustin Fliche, F-34295 Montpellier, Cedex 5, France; 5 Department of General and Liver Transplant Surgery, University of Montpellier, Saint Eloi Teaching Hospital, 80 avenue Augustin Fliche, F-34295 Montpellier, Cedex 5, France; 6 Department of Anesthesiology and Critical Care, University of Montpellier Saint Eloi Teaching Hospital, 80 avenue Augustin Fliche, F-34295 Montpellier, Cedex 5, France

## Abstract

**Introduction:**

Procalcitonin (PCT) biomarker is suggested to tailor antibiotic therapy in the medical intensive care unit (ICU) but studies in perioperative medicine are scarce. The aim of this study was to determine whether PCT reported thresholds are associated with the initial treatment response in perioperative septic shock secondary to intra-abdominal infection.

**Methods:**

This single ICU, observational study included patients with perioperative septic shocks secondary to intra-abdominal infection. Demographics, PCT at days 0, 1, 3, 5, treatment response and outcome were collected. Treatment failure included death related to the initial infection, second source control treatment or a new onset intra-abdominal infection. The primary endpoint was to assess whether PCT thresholds (0.5 ng/ml or a drop from the peak of at least 80%) predict the initial treatment response.

**Results:**

We included 101 consecutive cases. Initial treatment failed in 36 patients with a subsequent mortality of 75%. Upon admission, PCT was doubled when treatment ultimately failed (21.7 ng/ml ± 38.7 vs. 41.7 ng/ml ± 75.7; *P* = 0.04). Although 95% of the patients in whom PCT dropped down below 0.5 ng/ml responded to treatment, 50% of the patients in whom PCT remained above 0.5 ng/ml also responded successfully to treatment. Moreover, despite a PCT drop of at least 80%, 40% of patients had treatment failure.

**Conclusions:**

In perioperative intra-abdominal infections with shock, PCT decrease to 0.5 ng/ml lacked sensitivity to predict treatment response and its decrease of at least 80% from its peak failed to accurately predict treatment response. Studies in perioperative severe infections are needed before using PCT to tailor antibiotic use in this population.

## Introduction

Overuse of antibiotics is common in both medical and surgical (perioperative medicine) intensive care units (ICU) leading to the development of antimicrobial resistance and hospital-acquired infections [1]. To decrease hospital-acquired infection incidence, antimicrobial consumption reduction in the surgical ICU is needed but unfortunately controlled studies comparing two different durations of antibiotics are scarce [2]. So, strategies other than randomized controlled studies might be of interest [3,4]. Recently, new strategies to reduce antibiotic duration have included the development of biomarker-directed treatment algorithms [5,6]. Procalcitonin (PCT), the 116-amino acid precursor of calcitonin, is elevated consecutively in several systemic inflammatory conditions and its magnitude correlates well with injury severity and prognosis [7,8]. In the ICU, serial PCT measurement might be used as a surrogate to facilitate the early discontinuation of antimicrobials [5,9]. Indeed, it has been reported that using a PCT plasma threshold from 0.25 ng/ml to 0.50 ng/ml or its decrease of at least 80% compared to its peak [5,9-11] allows withholding antibiotics earlier without affecting clinical outcome [12-15]. Surprisingly, most of the studies that evaluated the interest of PCT to guide antimicrobial duration included mostly medical patients [5,9], although peritonitis is one of the most habitual reasons to admit a patient in a perioperative situation to the surgical ICU. To the best of our knowledge, few studies focused on PCT in peritonitis, with its very heterogeneous severity criteria [11,16,17].

As a first step to evaluate PCT as a tool to discontinue antibiotics, the aim of the present study was to assess whether the PCT thresholds of 0.5 ng/ml or its drop from the peak of at least 80%, previously reported in medical patients, could predict the response to initial treatment in surgical patients admitted for an intra-abdominal infection with septic shock. Our hypothesis is that the PCT kinetic may be associated with the patient’s response to the initial treatment.

## Material and methods

### Study setting and patients

This observational study was performed in an adult ICU of a university hospital from April 2008 to February 2011. Retrospective analysis was performed on data prospectively acquired from an electronic chart review that automatically records all physiological and biological data. Because of its observational, non-interventional design, the present study was approved by the local ethics committee (Comité d’Organisation et de Gestion de l’Anesthésie Réanimation du Centre Hospitalier Universitaire de Montpellier (COGAR)) and, in accordance with French law, informed consent was waived.

All consecutive patients (18 years or older) who were admitted to the ICU with abdominal septic shock or who developed septic shock consecutive to an intra-abdominal infection while hospitalized in the ICU were screened. In our ICU, it is part of our routine care to measure PCT levels upon ICU admission and subsequently every 48 to 72 h in case of septic shock. Patients discharged or dead before 48 h and those in whom PCT was not monitored for logistical reasons were not analyzed further. Patients admitted with acute pancreatitis were excluded because PCT is increased in acute pancreatitis, whatever the presence of an infectious complication [18].

### Definitions

Septic shock was defined by evidence of infection and a systemic response to infection, in addition to a systolic blood pressure of <90 mmHg, despite adequate fluid replacement, and a need for vasopressors for at least 1 hour, according to the American College of Chest Physicians/Society of Critical Care Medicine Consensus Conference Committee criteria [19]. Intra-abdominal infection was defined as an intra-abdominal septic focus requiring surgical treatment with proof of infection in the succeeding laparotomy or a documented intra-abdominal infection [20]. Successful treatment was defined by either an uneventful recovery (no further invasive procedures necessary) after the first line of treatment including invasive intervention or the absence of an intra-abdominal infectious focus at the time of relaparotomy if judged necessary [20-24]. As reported by other studies focusing on PCT [8] and/or peritonitis treatment response [11,16,21], treatment failure was defined by either death because of the initial infectious focus or a relaparotomy or radiological drainage showing the persistence of an ongoing intra-abdominal infection or a new onset intra-abdominal infection. We thus divided patients into a group with a successful initial treatment (treatment success group) and a group in which the initial treatment failed (treatment failure group).

### Treatment strategy

In all cases that needed surgery, peritoneal fluid was sampled for microbiology. After abundant peritoneal lavage, stomies were preferred to primary anastomosis but the attending surgeon in charge of the case made the final decision. Relaparotomy was exclusively performed on demand and not scheduled systematically. The patients received antibiotic therapy prior to anesthesia according to the Infectious Diseases Society of America (IDSA) guidelines [20]. For intra-abdominal infection with septic shock, we used piperacillin-tazobactam, amikacin and antifungal treatment when yeasts were positive on direct peritoneal fluid examination. The antibiotic therapy was continued until resolution of clinical signs of infection and recovery of gastrointestinal function according to the IDSA guidelines, but recommendation was made not to exceed 15 days if the patient’s condition improved [20].

### Baseline assessment and data collection

The following data were recorded upon ICU admission: demographic characteristics, microbiology on blood cultures, peritoneal and biliary tract samples, severity of underlying medical condition stratified according to the criteria of McCabe and Jackson, simplified acute physiology score II (SAPS II) [25], Sepsis-related Organ Failure Assessment (SOFA) score [26], the presence of co-morbidities, and reason for admission to the ICU. Microbiology data was also recorded. During the ICU stay, we collected the SOFA score at days 0, 1, 3 and 5 and the outcome including the need for relaparotomy, invasive procedure to complete the septic focus eradication, nosocomial infection occurrence, length of ICU stay, duration of mechanical ventilation and survival at ICU discharge. Septic focus cure was evaluated as defined above.

### Biomarkers: PCT and C-reactive protein (CRP) assays

For all patients, serum was collected for CRP and PCT assays upon admission (day 0) and subsequently at days 1, 3 and 5. For PCT, we used the previously published methodology [27]. Briefly, the biochemistry laboratory used TRACE (Time-Resolved Amplified Cryptate Emission) technology on a Kryptor analyzer (Brahms Diagnostica, Berlin, Germany). The Kryptor analyzer detection limit in 100 μl of serum was 0.019 ng/ml and sensitivity (interassay variation coefficient, 20%) was 0.06 ng/ml. The 95th percentile reference was 0.064 ng/ml.

### Endpoint

The primary endpoint was to assess whether most commonly reported PCT thresholds used to discontinue the antibiotics in the critically ill (either 0.5 ng/ml or the drop from its peak of at least 80%) could predict the response to the initial treatment (success vs. failure) in perioperative intra-abdominal infection with shock.

The secondary endpoints were the evaluation of the relation between the PCT kinetic and other markers (temperature, CRP and SOFA) kinetics with response to treatment.

### Statistical analysis

Data are expressed as mean ± SD or SEM for normally distributed data, and median with interquartile range (IQR) for non-normally distributed data. Continuous variables were compared using Student’s *t* test for normally distributed variables and the Mann-Whitney rank-sum test for non-normally distributed variables. The chi-square test or the Fisher exact test was used to compare categorical variables. Because the kinetic of biomarkers was more evaluated than their absolute values in the present study, we used a mixed logistic regression model taking into account both time (as repeated measures were performed and analyzed) and biomarkers in the comparison model. Biomarker and time were considered as fixed effects and outcome (success vs. treatment failure) was considered as the dependent variable. Comparisons were performed between day 0 and day 5. Missing biomarker data were neither deleted nor replaced or imputed as missing data are handled by the mixed logistic regression [28]. Sensibility, specificity, positive and negative predictive values as well as accuracy for both thresholds (PCT decrease below 0.5 ng/ml or drop from its peak of at least 80%) were also calculated. Statistical analysis was performed by an independent statistician (NM), with R software (version 2.10.1). Significance was established at *P* <0.05.

## Results

**Figure 1:**
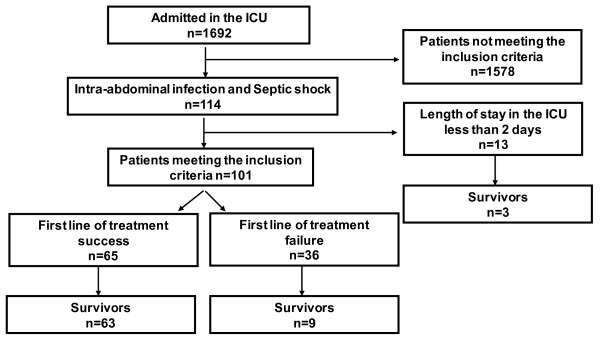
Flow chart of the study.

During the study period, among the 1,692 patients admitted to our ICU, 101 consecutive patients meeting the inclusion criteria were included (Figure 1). Patients who were discharged within two days after ICU admission were not analyzed further (Figure 1). Patients’ demographics are presented in Table 1. Surgical procedure was necessary in 87% of the patients and all were treated with vasopressors and mechanical ventilation within the first 24 hours of ICU stay. Microbiology culture is presented in Table 2. No differences were noted according to the treatment response. Initial treatment failed in 36 patients (Table 3). The main cause of failure was relaparotomy or radiological drainage for additional source control in 17 cases (47%) or death related to the initial infection in 14 cases (39%). Four patients in the treatment success group needed a relaparotomy or a radiological drainage for hematoma (n = 2) or a superinfection suspicion (n = 2) but no infection was diagnosed by this second look. Antibiotic spectrum modification because of treatment failure or nosocomial infection occurred in 14 patients in the treatment failure group (39%) vs. 5 in the treatment success group (8%), *P* = 0.003. At day 28, treatment response was significantly associated with mortality (Table 3).

**Table 1:** Characteristics of the study population

	**All**	**Treatment success**	**Treatment failure**	***P***
	**(n = 101)**	**(n = 65)**	**(n = 36)**	
Age (years),	66 ± 15	66 ± 16	66 ± 12	0.60
Male	60 (60)	40 (62)	20 (56)	0.71
Body mass index (kg/m^2^)	27.1 ± 7.5	26.2 ± 5.2	28.2 ± 10.3	0.14
SAPS II upon ICU admission	49 ± 17	46 ± 15	56 ± 17	0.006
SOFA upon ICU admission	9.7 ± 3.2	9.0 ± 2.6	11.1 ± 3.3	0.0003
Respiratory	1.8 ± 1.0	1.6 ± 1.0	2.2 ± 0.9	<0.01
Hemodynamic	4.0 ± 0	4.0 ± 0	4.0 ± 0	>0.99
Neurologic	1.8 ± 0.9	1.8 ± 0.9	2.1 ± 1.0	0.09
Liver	0.8 ± 1.1	0.6 ± 0.8	1.1 ± 1.4	0.08
Hematology	0.6 ± 0.9	0.5 ± 0.9	0.7 ± 0.8	0.05
Kidney	1.0 ± 1.1	0.8 ± 1.0	1.3 ± 1.1	0.04
Past medical history				
Hypertension	45 (45)	27 (42)	18 (50)	0.84
Coronary artery disease	20 (20)	13 (20)	7 (19)	0.94
NYHA III-IV heart insufficiency	14 (14)	8 (12)	6 (17)	0.56
COPD	13 (13)	8 (12)	5 (14)	0.82
Diabetes mellitus	21 (21)	14 (22)	7 (19)	0.80
Cancer	40 (40)	26 (40)	14 (39)	0.82
Cirrhosis	12 (12)	7 (11)	5 (14)	0.64
Site of septic focus				
Distal esophagus/stomach	14 (14)	8 (12)	6 (17)	0.54
Biliary tract	20 (20)	13 (20)	7 (19)	0.95
Small intestine	26 (26)	18 (28)	8 (22)	0.54
Colorectal	36 (36)	25 (38)	11 (31)	0.43
Spontaneous peritonitis	2 (2)	0	2 (6)	0.06
Other	3 (3)	1 (2)	2 (6)	0.25
Surgical procedure performed	87 (87)	58 (89)	29 (81)	0.23

Categorical data are expressed as number and percentage. Continuous data are expressed as mean and standard deviation or median and quartiles. Comparisons were made between patients in whom the first-line treatment succeeded and patients in whom the first-line treatment failed. *COPD,* chronic obstructive pulmonary disease; *ICU,* intensive care unit; *NYHA,* New York Heart Association; *SAPS II,* severity acute physiology score II [23]: *SOFA,* sequential organ failure assessment [24].

**Table 2:** Etiology of the intra abdominal infection

**Microorganisms**	**All**	**Treatment success**	**Treatment failure**	***P***
	**(n = 134)**	**(n = 89)**	**(n = 45)**	
*E. coli*	35 (26)	21 (24)	14 (31)	0.35
*Enterobacter sp*	11 (8)	7 (8)	4 (9)	0.83
*P. aeruginosa*	8 (8)	6 (7)	2 (4)	0.96
*E. faecalis*	12 (12)	7 (8)	5 (11)	0.54
*E. faecium*	13 (9)	8 (9)	5 (11)	0.69
Other *Enterobacteriaceae*	17 (13)	14 (16)	3 (7)	0.14
*Candida sp*	11 (11)	7 (8)	4 (9)	0.84
Anaerobes	8 (6)	3 (4)	5 (11)	0.07
Others	19 (14)	16 (18)	3 (7)	0.07

Categorical data are expressed as number and percentage.

**Table 3:** Outcome characteristics of the 101 patients according to the initial treatment response

	**All**	**Treatment success**	**Treatment failure**	***P***
	**(n = 101)**	**(n = 65)**	**(n = 36)**	
Relaparotomy needed	21 (21)	3 (5)	18 (50)	<0.001
Surgical drainage after first line treatment	8 (8)	1 (1)	7 (19)	0.001
Duration of antibiotic treatment during ICU stay (days)	11.7 ± 7.2	11.3 ± 6.1	12.9 ± 8.8	0.84
Nosocomial infection	36 (36)	15 (23)	21 (58)	<0.001
Cytomegalovirus reactivation	5 (5)	2 (3)	3 (8)	0.24
Duration of mechanical ventilation (days)	8 ± 6	6 ± 7	9 ± 9	0.002
ICU length of stay (days)	11 ± 10	10 ± 9	14 ± 11	0.08
Mortality in the ICU	29 (29)	2 (3)	27 (75)	<0.001
Cause of death				
Refractory shock related to initial infection	5 (5)	0	5 (14)	<0.001
Secondary surgical complication	7 (7)	0	7 (19)	<0.001
Nosocomial infection as the main cause of death	4 (11)	0	4 (11)	<0.001
Other including intensive care withdrawal	13 (13)	2 (3)	11 (31)	<0.001

Continuous data are expressed as mean and standard deviation. Categorical data are expressed as number and percentage. *ICU,* intensive care unit.

**Figure 2:**
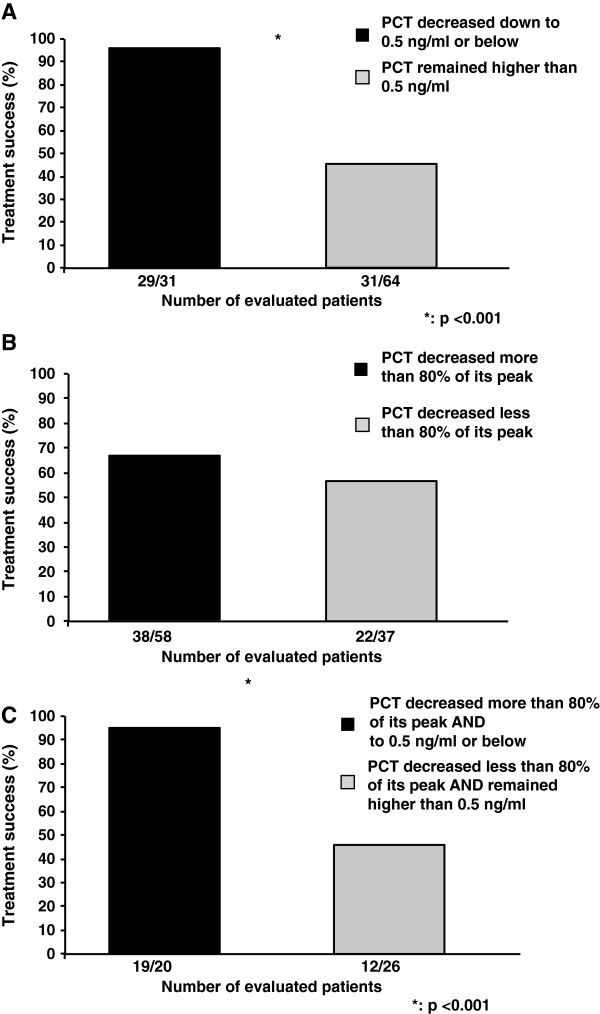
**Treatment response according to procalcitonin drop. (A)** Percentage of treatment success according to the lowest PCT value from ICU admission to day 5. Patients were dichotomized according to whether the lowest PCT value was inferior to 0.5 ng/ml or not. Among the 31 patients in whom PCT decreased below the threshold of 0.5 ng/ml, 29 responded successfully to the treatment. Thirty-one patients among 64 did also respond successfully to treatment although PCT remained superior to 0.5 ng/ml. **(B)** Percentage of treatment success according to PCT decrease from its peak value from ICU admission to day 5. Patients were dichotomized according to whether the PCT value decreased by more than 80% of the peak value or not. Among the 58 patients in whom PCT decreased by at least 80% from its peak, 38 responded successfully to the treatment. Twenty-two patients among 37 did also respond successfully to treatment although PCT drop was lower than 80% of the peak. **(C)** Percentage of treatment success according the lowest PCT value and the PCT decrease from its peak value from ICU admission to day 5 (combination of **(A)** and **(B)**). Among the 20 patients in whom PCT both decreased by at least of 80% from its peak and below 0.5 ng/ml, 19 responded successfully to the treatment. Twelve patients among 26 did also respond successfully to treatment although PCT drop was lower than 80% of its peak and its absolute value remained superior to 0.5 ng/ml. On the x-axis is presented the number of patients in whom the treatment was successful of the number of patients analyzed. Six patients could not be analyzed because of logistical reasons.

Upon ICU admission, PCT in patients with subsequent failure of initial treatment was double than in those with successful initial treatment, *P* = 0.04. We assessed whether the PCT thresholds of either 0.5 ng/ml or its relative drop of at least 80% from the peak could predict the treatment response. Among the 101 patients, six patients (five in the success and one in the failure group) had less than three PCT measurements for logistical reasons and could therefore not be analyzed adequately. Almost 50% of cases responded successfully to treatment even if PCT concentration was constantly above 0.5 ng/ml during the ICU stay (Figure 2A). A decrease of at least 80% compared to the peak was not associated with treatment success (Figure 2B). When PCT decreased below 0.5 ng/ml and its drop was equal to or greater than 80% of its peak, 95% did respond successfully to treatment. Interestingly, when PCT decrease remained superior to 0.5 ng/ml and above 80% of its peak, almost 50% of the patients also responded successfully to the initial treatment (Figure 2C). Using the threshold of 0.5 ng/ml, PCT predicted the treatment success with a sensitivity of 48%, specificity of 94%, positive predictive value of 94%, negative predictive value of 52% and accuracy of 65%. Using a drop equal to or greater than 80% of the peak, PCT predicted the treatment success with a sensitivity of 63%, specificity of 43%, positive predictive value of 66%, negative predictive value of 41% and accuracy of 56%. Among the 35 patients admitted for a post-operative intra-abdominal infections, we found similar performance values for PCT without significant differences compared to patients admitted for community-acquired intra-abdominal infection.

**Figure 3:**
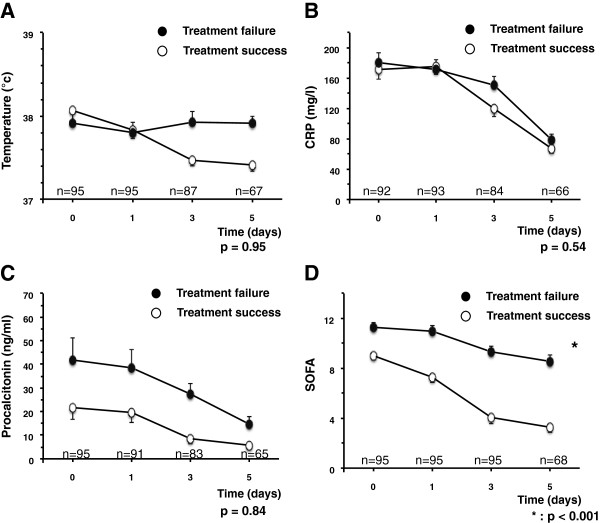
**Treatment response according to temperature, C-reactive protein, serum procalcitonin and SOFA score kinetics.** Kinetics of temperature **(A)**, C-reactive protein **(B)**, serum procalcitonin **(C)** and Sepsis-related Organ Failure Assessment (SOFA) score **(D)** in patients according to the initial treatment response from day 0 to day 5. Comparisons were made to assess whether the biomarkers kinetic and not their absolute values are different according to the initial treatment impact between day 0 and day 5. Results are expressed as means ± standard error for the mean (SEM). On the x-axis is presented the number of patients in whom data were available.

However, when the PCT kinetic between admission and day 5 was considered, it was not significantly different according to treatment response (Figure 3).

We then examined the relation between temperature, CRP and SOFA kinetics and the treatment response. Neither temperature nor CRP drops between admission and day 5 were different according to the patient’s response to treatment (Figure 3A and 3B). Interestingly, SOFA score drop was superior in patients who responded successfully to the treatment compared to patients who did not (*P* <0.001) (Figure 3D).

## Discussion

The present study reports that neither PCT threshold of 0.5 ng/ml nor its decrease of at least 80% from its peak value could accurately predict the treatment response in a subpopulation of intra-abdominal cases with septic shock. Although 95% of patients in whom PCT decreased below 0.5 ng/ml responded successfully to treatment, 50% of the patients in whom PCT remained superior to 0.5 ng/ml also responded positively to the treatment making this threshold of 0.5 ng/ml a specific but not a sensitive biomarker. Decrease of at least 80% from the peak was not associated with treatment response. In perioperative medicine, PCT may be used to tailor antibiotic therapy using either an absolute cutoff (for example 0.5 ng/ml) or a significant drop compared to the peak value (for example 80%) [12]. In the present study, we examined whether those thresholds were associated with initial treatment response in perioperative critically ill patients admitted for an intra-abdominal infection with shock.

PCT was initially used as a diagnostic biomarker. However, high interindividual differences, failure of a single measurement to accurately identify infection [27,29] and false positive cases have been reported [30]. Recent meta-analyses and reviews concluded that PCT cannot reliably differentiate infectious from noninfectious causes of inflammation in critically ill patients [30]. Moreover, PCT threshold to differentiate bacterial infection vs. inflammation is commonly higher in perioperative medicine than in medical patients [31]. Another way to consider PCT interest in critically ill patients is to use it as a guide to discontinue the antibiotic therapy earlier. Studies that focused on PCT as a tool to withdraw the antibiotics earlier used both an absolute value of PCT (from 2 to 0.25 ng/ml) and a significant drop from the peak value (either a significant reduction of 25 to 35% in three to five days or a reduction of up to 90% compared to the peak) [5,7,9-11,32]. In the present study, we studied, as recently suggested [9,12] whether an absolute drop down to at least 0.5 ng/ml or a significant reduction of at least 80% compared to the peak value was associated with the success of the initial treatment for septic shock related to an intra-abdominal infection. We reported that a PCT threshold of 0.5 ng/ml was specific but neither sensitive nor accurate and that a PCT decrease of at least 80% from its peak was not associated with the patient’s response to treatment (Figures 2, 3). Duration of antibiotics in this subpopulation could not be recommended based on PCT level whatever the threshold used.

This finding contrasts with studies on ventilator-associated pneumonia prognosis [8] and with the main study that focused on the PCT kinetic as a prognostic biomarker in nonselected critically ill patients. However, those studies focused on pneumonia or on medical patients, including less than 20% of patients needing surgery although higher PCT ranges might be observed in surgical patients [31].

Studies focusing on the PCT static threshold value (for example 0.5 ng/ml) to predict the patient’s outcome in severe intra-abdominal infections are sparse. In a study combining 246 cases of sepsis, severe sepsis or septic shock secondary to peritonitis, PCT was associated with patient’s survival in the ICU [17]. Conversely, another study reported that PCT of 16 ng/ml discriminated survivors vs. nonsurvivors with a positive predictive value of only 30% in secondary peritonitis [16]. To the best of our knowledge, the present study is the first to focus on a homogenous group with all patients presenting septic shock related to an intra-abdominal infection. We report that, upon admission, the PCT absolute value might be an indicator of treatment failure (Figure 3) but a PCT decrease of at least 80% from the peak or its kinetic from admission to day 5 could not accurately predict treatment response (Figure 2B). One study reported that PCT but not APACHE II day 2/day 1 ratio was associated with the treatment response in a case mix with an unknown ratio of shock [21]. Another single center study prospectively included 37 patients (including 21 pneumonia cases) with a PCT peak value above 10 ng/ml [33]. The authors reported that the PCT kinetic between day 0 and day 5 was not better than the SOFA kinetic to predict ICU mortality. We focused on initial treatment response rather than on mortality but our results are consistent with that study, highlighting that organ failure score (SOFA score in the present study) might be as helpful as other biomarkers to assess response to treatment in critically ill patients.

The present study has some limits. First, it is a single-center observational study. Second, to distinguish treatment failure from treatment success, we considered that failure of treatment could be either deaths related to infection or infectious surgical complications. Although it may appear cumbersome to cumulate death with other complications, these criteria have been extensively used in studies on antibiotic evaluation. Patients with a length of stay in the ICU of less than two days were not analyzed to avoid fulminant septic shock. Third, we did not perform receiver operating characteristic curves as the aim of the present study was to assess whether published thresholds could be used in intra-abdominal infection with shock rather than to determine new thresholds in another population of interest. Finally, PCT could remain persistently above 0.5 ng/ml because of other reasons than abdominal infection but treatment response was assessed based solely on the abdominal infection course.

## Conclusions

In this cohort of 101 perioperative cases with septic shock consecutive to an intra abdominal infection, we report that, PCT threshold of 0.5 ng/ml or its decrease of at least 80% from its peak are not accurate markers to predict patient’s response to the initial treatment. Before using PCT to discontinue antibiotic therapy in surgical patients, further studies evaluating specifically PCT in surgical septic shock are needed.

## Key messages

• Procalcitonin decrease below 0.5 ng/ml was specific but neither sensitive nor accurate to predict treatment response in 101 septic shock patients secondary to an intra abdominal infection

• Procalcitonin decrease of at least 80% from its peak failed to accurately predict treatment response in this population

## Abbreviations

CRP: C-reactive proteine; ICU: Intensive care unit; IDSA: Infectious diseases society of America; PCT: Procalcitonin; SAPS II: Simplified acute physiology score II; SOFA: Sepsis-related organ failure assessment.

## Competing interests

Boris Jung has received speaking fees from Merck, not in relation with the present study. Samir Jaber has received research grants and speaking fees from Maquet, Draeger, Hamilton Medical, Fisher Paykel and Abbott, not in relation with the present study. The other co-authors declare they have no competing interests.

## Authors’ contributions

BJ, SJ and GC designed the study, analyzed the results and wrote the manuscript. MN and ZH collected data and analyzed the results. HJP analyzed the results and helped in the interpretation of the microbiological data. FP made corrections to the manuscript and surgical thoughts. NM analyzed the results and performed the statistical analysis. All authors read and approved the final manuscript.
